# Making the invisible visible: maternal and child health inequalities for Latin Americans in the UK

**DOI:** 10.1016/j.lanepe.2026.101805

**Published:** 2026-08-11

**Authors:** Cristina Fernandez Turienzo, Maria Alvarez Gomez, Luana Barbagelata, Jacobo Belilty, Bruna Boscaini, Alex Hernandez, Rose Higgins, Joseph Lam, Ramon Luengo Fernandez, Diana Medina, Judith Rankin, Rubi Rodriguez Nieto, James Scuffell, Octavia Wiseman, Jane Sandall

**Affiliations:** a Department of Women & Children's Health, School of Life Course & Population Sciences, Faculty of Life Sciences & Medicine, King's College London, UK; b Latin American Women’s Rights Service, UK; c Indoamerican Refugee and Migrant Organisation, UK; d Coalition of Latin Americans in the UK, UK; e Latin Americans in Health and Care UK Network, UK; f Nuffield Department of Primary Care Health Sciences, University of Oxford, UK; g Population, Policy and Practice Department, University College London, UK; h National Perinatal Epidemiology Unit, University of Oxford, UK; i King’s College Hospital NHS Foundation Trust, UK; j Population Health Sciences Institute, Newcastle University, UK; k Latin American Migrant Maternity Network, UK; l Department of Primary Care and Data Science, King’s College London and Minet Green Health Practice, London, UK; m Centre for Maternal and Child Health Research, City St. George’s, University of London, UK

The Latin American community—encompassing individuals with heritage from the Spanish- and Portuguese-speaking countries of the Americas and the Caribbean, and identified by this inclusive term rather than the US-origin classification ‘Hispanic’ which excludes Brazilians, implies an Iberian rather than postcolonial identity, and flattens the population’s rich Indigenous, Afro-Latin, and mestizo diversity—is one of the fastest-growing ethnic groups in the United Kingdom, yet remains “statistically invisible” in health policy and data.^1^ While 2021 Census data identifies over 280,000 residents born in Latin American countries,^2^ the actual population size, including second-generation and undocumented migrants, is likely closer to 500,000.^1^ Nationally, this group is already comparable in size to the British Asian population of 1991. While London remains the primary hub, the population is expanding nationally with large populations in Liverpool and the West Midlands, which reported a 124% increase in Latin American-born residents between 2010 and 2021.^3^ Despite their presence, “Latin American” is not yet a standard ethnic category in NHS or Office for National Statistics (ONS) data systems, though it was the second largest write-in response within the 2021 Census ‘Other’ category.^2^ Consequently, forced identification as ‘Other’ or ‘White Other’ hides their health needs, even though these women give birth to over 5000 infants annually in the UK.^4^

While UK-specific data on maternal and perinatal outcomes is limited, international evidence points to important disparities among Latin Americans. A meta-analysis in The Lancet, which analysed over two million pregnancies across 20 high-income countries, found that babies born to women categorised as Hispanic (defined in this review as women in the USA of Spanish-speaking or Latin American descent or heritage irrespective of their racial identity) had three-times the risk of neonatal death (death within the first 28 days) compared to those born to White women.^5^ In contrast to Black women who consistently had worse outcomes (including stillbirth, preterm birth, small for gestational age), neonatal mortality was the primary increased risk identified for Hispanic women.^5^ Although these findings are derived largely from US populations and may not be directly generalisable to the UK, they signal a potential problem for routine surveillance, particularly as this differential in neonatal death is almost double that identified in the UK between babies born to Black and White women.^6^

We cannot address health inequities we do not measure; without routine ethnic classification, maternal and perinatal disparities cannot be monitored or addressed. In the UK, Latin Americans face intersecting risks that delay service engagement and directly affect maternal safety, quality, and care experiences. Many have undocumented or precarious status or no recourse to public funds. Fear of NHS charging policies and potential data-sharing with enforcement agencies creates a hostile environment that drives late antenatal booking and poor engagement with services.^7^ Only 38% of migrant women in vulnerable circumstances receive their first antenatal appointment by 12 weeks, and 50% attend fewer than the recommended visits.^8^ Perceived surveillance and discrimination, low-paid employment with unsociable hours, severe language barriers, and unfamiliarity with the health and social care systems (eg referral process, GP online access) lead to practical exclusion from services.^1^^,^^7^^,^^9^ Delayed access to care is layered upon higher baseline rates of multiple long-term conditions, including diabetes and cardiovascular disease,^10^ alongside specific risks such as Chagas disease (which can be vertically transmitted from mother to child)^11^ and potential underdiagnosis of HIV.^12^ Driven by these intersecting layers of disadvantage, Latin American women frequently adopt transnational health-seeking strategies, including obtaining medication from their countries of origin or informal networks.

While extensive research, including the Lancet series on Racism and Health and the forthcoming Commission on Racism and Child Health, highlight how structural racism drives maternal and child health disparities for Black and Asian women, the specific outcomes for Latin American women in the UK remain unexplored.^13^ Existing European literature is heavily skewed towards Spain, leaving a critical knowledge gap regarding health determinants and care costs in other countries including the UK.^14^ As emphasized by recent health policy reports, such as the King’s Fund, women from ethnic minority groups are not a homogenous population and “blanket” approaches fail to address the specific needs of distinct communities. For the Latin American community, structural racism, economic deprivation, and precarious legal status intersect to create profound barriers to safety. However, designing inclusive services is contingent on the ability to measure outcomes, a task currently impossible for this population. To close this gap, simultaneous policy action and targeted research are required. The foundational requirement is structural visibility through the formal adoption of Latin American as a distinct ethnic category by the ONS and within NHS data systems, building on the consultation momentum submitted by community networks in February 2026. In parallel, NHS England’s Ethnicity Recording Improvement Plan provides an opportunity for Integrated Care Boards and healthcare providers to improve the granularity of local recording practices and reduce reliance on residual ‘Other’ categories. Moving forward, we must work alongside community groups and organisations to ensure this ethnicity becomes a standard metric and enables the assessment of health needs, outcomes and experiences of Latin American population, including women and their babies, and co-produce targeted, community-driven solutions, including peer support and advocates, which have been shown to significantly improve pregnancy outcomes and service engagement. Until Latin American ethnicity is routinely measured, disparities in maternal and child health will remain hidden and beyond the reach of effective policy action; visibility is the first step towards equity.

## Contributors

CFT drafted the initial draft and all authors (MAG, LB, JB, BB, AH, RH, JL, RLF, DM, JR RRN, JScu, OW, JS) commented and approved the final manuscript.

## Declaration of interests

JScu is an elected committee member of Lambeth Local Medical Committee. Other authors declare no competing interests.
